# Health Development versus Medical Relief: Not a Turf Battle

**DOI:** 10.1371/journal.pmed.0030508

**Published:** 2006-11-28

**Authors:** Guy-André Pelouze

**Affiliations:** St. John Hospital, Perpignan, France

Médecins Sans Frontières is a respectable organisation which helps people in jeopardy and provides medical relief in difficult situations all over the world. Despite this recognition I must confess I found the last paper of G. Ooms in *PLoS Medicine* [1] to develop highly debatable concepts about health, development, and even sustainability. Ooms postulates that sustainable intervention creates a bias for development agencies to maintain the status quo.

On the other hand, it could very easily be argued that conventional emergency “humanitarian aid” has failed to improve health on even a medium-term basis in the last four decades. Common sense indicates that populations benefit more from improvements in wealth, water supply, and agriculture than from consuming free goods from international aid.

Several studies provide a basis for this conclusion [2]. On the contrary, a persistent status quo could result from an exclusive humanitarian approach, precluding the necessary changes to develop health care.

International agencies say that, based on numerous studies, improving health on a medium- or long-term basis is a matter of “sustainable” programmes. But what is meant by “sustainability”? It seems that Dr. Ooms interprets sustainability as durability, a confusion which is obvious when one reads the French version of his paper. Durability is a plain matter of time; pollution, totalitarian regimes, or poverty could as well, unfortunately, meet a single durability criterion. This is the main point, if one wants to assess the criticism which Ooms aims at development agencies. Their main objective is to assess the sustainability of the health programmes which means (in French the *supportabilité* and not the *durabilité*) whether these programs exceed the economic, organisational, and ecological possibilities of the country and its population. And I recognize that I am in keeping with that! To give rice to people who usually consume rice is helpful, but next time it will be even more helpful to give them the means to grow their own!

Yet the reality is far more complex, something which requires that agencies carefully assess the different programmes [3]. I must add that such an approach does not preclude an increase in annual governments outlays. But before increasing expenditure, it is wise to assess whether the programmes is working, and for whom. I don't miss the point that sustainable aid is for certain governments synonymous with conditional aid, and such difficulties must not be hidden, but every one of us is able to make the distinction between the principle and some penny-pinching, restrictive policies which can be amended and reversed.

Indeed, the two approaches are complementary. When an emergency situation arises, it is obvious that some of the critical health issues of the local populations could be addressed by emergency international aid. But after a few weeks, only structural and political changes (that is, peace, convenient water supply, agriculture revival, affordable energy, information, free trade, free enterprise through microrenting...) are crucial for maintaining health and eventually improving it. As a matter of fact it could be a more dangerous illusion for these endangered populations to give credit to the ideas that Dr. Ooms develops in order to justify the spending of more public funds to extend emergency humanitarian aid indefinitely.
